# Characteristics and Antitumor Activity of *Morchella esculenta* Polysaccharide Extracted by Pulsed Electric Field

**DOI:** 10.3390/ijms17060986

**Published:** 2016-06-22

**Authors:** Chao Liu, Yonghai Sun, Qian Mao, Xiaolei Guo, Peng Li, Yang Liu, Na Xu

**Affiliations:** 1 College of Food Science and Engineering, Jilin University, Changchun 130000, China; liuchaocarrol@163.com (C.L.); mianfeiyouxiangmqq@163.com (Q.M.); gina_guoxiaolei@163.com (X.G.); m13704323904@163.com (Y.L.); doctorserena@163.com (N.X.); 2 School of Food Engineering, Jilin Agriculture Science and Technology College, Jilin 132101, China; lipeng8098@126.com

**Keywords:** polysaccharide, *Morchella esculenta*, chemical structure, anti-proliferating activity, pulsed electric field

## Abstract

Polysaccharides from *Morchella esculenta* have been proven to be functional and helpful for humans. The purpose of this study was to investigate the chemical structure and anti-proliferating and antitumor activities of a *Morchella esculenta* polysaccharide (MEP) extracted by pulsed electric field (PEF) in submerged fermentation. The endo-polysaccharide was separated and purified by column chromatography and Gel permeation chromatography, and analyzed by gas chromatography. The MEP with an average molecular weight of 81,835 Da consisted of xylose, glucose, mannose, rhamnose and galactose at the ratio of 5.4:5.0:6.5:7.8:72.3. Structure of MEP was further analyzed by Fourier-transform infrared spectroscopy and ^1^H and ^13^C liquid-state nuclear magnetic resonance spectroscopy. Apoptosis tests proved that MEP could inhibit the proliferation and growth of human colon cancer HT-29 cells in a time- and dose-dependent manner within 48 h. This study provides more information on chemical structure of anti-proliferating polysaccharides isolated from *Morchella esculenta*.

## 1. Introduction

Wild and artificial mushrooms are valued by humans as an edible and medical resource, as they are rich in essential bio-macromoleules, such as polysaccharides, proteins and polynucleotides. In recent years, some bioactive polysaccharides isolated from medicinal mushrooms have attracted much attention from the fields of biochemistry and pharmacology. Some mushroom extracts have promising therapeutic effects on cardiovascular diseases, cancers, diabetes [1], and colon cancer, which is one of the causes of cancer mortality [2].

A variety of medicinal mushrooms (e.g., *Morchella esculenta* (L.) Pers. (*M. esculenta*)), which are traditionally utilized both as dietary supplements and for cancer treatment, have become increasingly popular [3].

*M. esculenta* is cherished for both nutritional and medicinal values because of the possession of many bioactive substances, including polysaccharides, proteins, trace elements, dietary fibers and vitamins [4]. *M. esculenta* has been proven to have anti-inflammatory and antitumor activities [5,6], which were attributed to the possession of polysaccharides [7]. Since *M. esculenta* is a rare wild resource and its cultivation via traditional methods is very impractical, submerged fermentation was introduced as an alternative. Some polysaccharides extracted from *M. esculenta* mycelia are potentially tumor-resistant [8]. 

Because of water-solution, most functional polysaccharides could be isolated through water extraction assisted with physical methods. However, some physical methods such as radiation means (e.g., heating, microwave and power ultrasound) would cause serious degradation of polysaccharides, reflected especially in their molecular weights (*M*_W_). Many functional properties of polysaccharides depend on the molecular mass [9]. Thus, in this study, we selected pulsed electric field (PEF) treatment, which is outstanding for having a significantly higher extraction yield and shorter extraction period [10]. In addition, PEF is a non-thermal technology [11] for the extraction of active ingredients from natural biomaterials unlike the conventional extraction method. PEF extraction can be performed at room temperature without any heating process, except for temperature rise resulting naturally, even at moderate electric field intensity of 30 kV·cm^−1^ [12]. However, the *M*_W_ of polysaccharides rapidly decreased by nearly 50% after ultrasonic treatment [9,13], and even more after microwave extraction [14].

There is little literature regarding the extraction of anti-proliferating and antitumor polysaccharides from *M. esculenta*, and a common problem mentioned in the existing articles is thermal degradation. In order to identify anti-proliferating polysaccharides for tumor treatment, we used PEF to isolate *M. esculenta* polysaccharides (MEP). Some MEP fractions were purified and elementarily characterized in order to find out if any fraction(s) have potential cytotoxic effect on colon cancer cells. The aim of this study was therefore to validate whether PEF treatment could extract endo-polysaccharide(s) from *M. esculenta* with little thermal degradation.

## 2. Results

### 2.1. Optimization of Pulsed Electric Field (PEF) Extraction

According to the experimental conditions and corresponding response shown in Table 1, the models for PEF extraction were determined from the following quadratic prediction function.

(1) $$\text{MEP Yield}=53.28-3.23\times X_{1}+3.46\times X_{2}-3.23\times X_{3}-2.75X_{1}X_{2}+2.74\times X_{1}X_{3}-1.26\times X_{2}X_{3}-6.29\times X_{1}^{2}-6.65\times X_{2}^{2}-6.66\times X_{3}^{2}$$

The results of the model in the form of analysis of variance (ANOVA) were given in Table 2. According to the *F*-value (27.81) and *p*-value (0.0001), the second-order model was significant between dependent variables and MEP yield (*p* < 0.05). ANOVA showed a good model performance with the correlation coefficient (*R*^2^) of 0.9725. The lack of fit in the model was not significant (*p* = 0.6673), which also indicated the accurate prediction of the model. As shown in Table 3, the reaction factors of X_1_ electric field intensity (*p* = 0.0022), X_2_ pulse number (*p* = 0.0015), X_3_ material-to-liquid ratio (*p* = 0.0022), X_1_X_2_ (*p* = 0.0252) and X_1_X_3_ (*p* = 0.0256) were all significant in this model. The predicted optimum PEF extraction condition calculated from the regression equation is as follows: X_1_ = 17.90 kV·cm^−1^, X_2_ = 6.76, and X_3_ = 1:26.35 g·mL^−1^. Under this condition, the predicted MEP yield was maximized to 55.21 µg·mL^−1^. In practice, the operated condition is X_1_ = 18 kV·cm^−1^, X_2_ = 7 and X_3_ = 1:27 g·mL^−1^, with the MEP yield of 56.03 µg·mL^−1^, which was consistent with the prediction (55.21 µg·mL^−1^).

**Figure 1:**
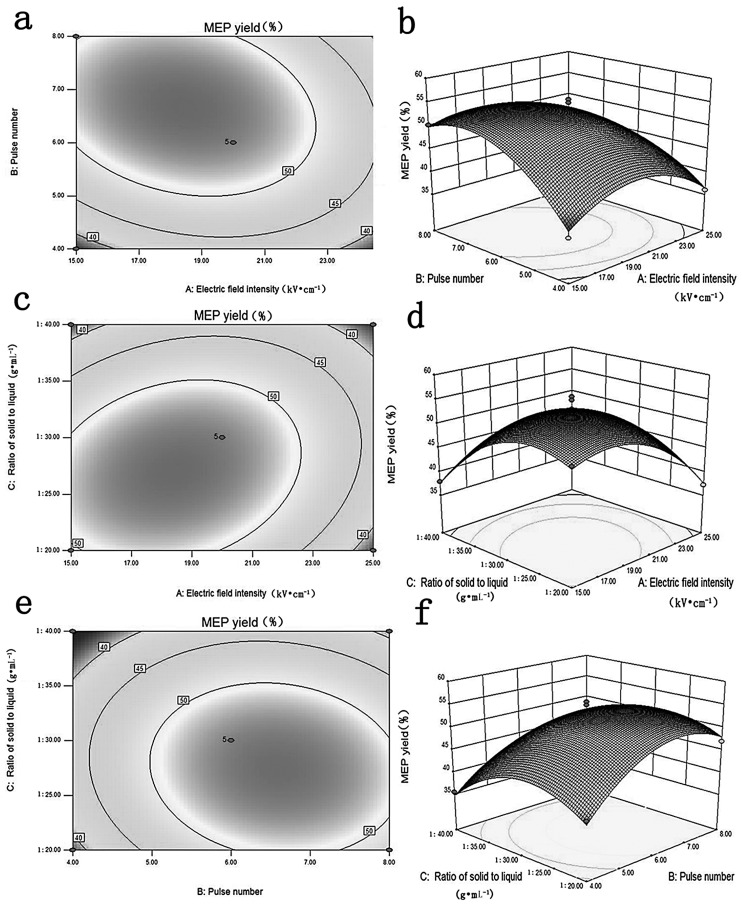
The effects of electric field intensity (X_1_), pulse number (X_2_) and material-to-liquid ratio (X_3_) on *M. esculenta* polysaccharide (MEP) yield of 3D response surface curves (**a**,**c**,**e**) and contours (**b**,**d**,**f**).

Figure 1 showed the combined effects of X_1_, X_2_ and X_3_ on MEP yield. The MEP yield was maximized to 55.62 µg·mL^−1^ at X_1_ = 20 kV·cm^−1^ and X_2_ = 6 while X_3_ was controlled under 1:30 g·mL^−1^ (Figure 1a,b). The quadratic effects of X_1_ and X_3_ on MEP yield are shown in Figure 1c,d, in which the MEP yield increased first and then declined. The same quadratic effect of X_2_ and X_3_ was found in Figure 1e,f. These results clearly reconfirm the maximum MEP yield calculated from the regression equation. The optimal conditions of PEF ensure the maximization of MEP yield.

### 2.2. Seperation and Purification

**Figure 2:**
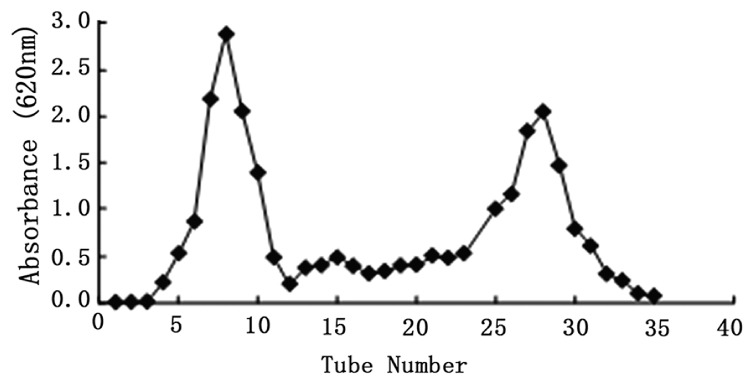
Purification of crude MEP by column chromatography.

Column chromatography results of MEP are shown in Figure 2. Two symmetric peaks of polysaccharides were detected by colorimetry and the corresponding fractions (F1 and F2) were collected by elution. F1 was found with a high yield as well as large *M*_W_ identified by gel permeation chromatograph (GPC) later. The tubes for F1 (#5–10) were combined, and the tubes for F2 (#25–30) were combined separately). Then, F1 and F2 were lyophilized.

### 2.3. Determination of M_W_s of Morchella esculenta Polysaccharide (MEP)

**Figure 3:**
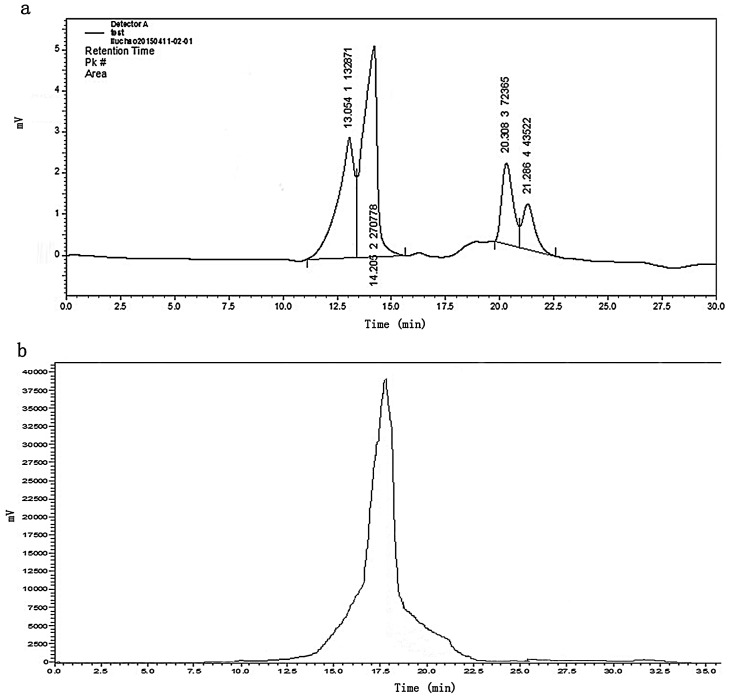
High-performance liquid chromatography (HPLC) spectra of: F2 (**a**); and purified M2 (**b**).

The average *M*_W_s of both F1 and F2 were calculated via GPC. The average *M*_W_ of F1 was over 2 × 10^6^ Da. As reported, polymers could modulate cell growth in an *M*_W_-dependent way [15]. For certain applications, particularly in the field of medical treatments, low-*M*_W_s polysaccharides have been selected over high *M*_W_s candidates because of their improved diffusion into biological tissues. F2 with lower average *M*_W_s was proven to be anti-proliferative in accordance with the above viewpoint. Then, F2 was further divided into four fractions (M1, M2, M3 and M4) (Figure 3a) with mean *M*_W_s of 222,344, 81,835, 428 and 129 Da, respectively, and analyzed by high-performance liquid chromatography (HPLC) (Table 3). F2 was mainly composed of M1 (25.575%) and M2 (52.119%). M3 and M4 with low *M*_W_s were predicted to not be significant for further research. Since the peaks of M1 and M2 on the chromatogram overlapped, we collected only the top fraction. However, the yield of M1 was too little for further study, while the second peak of M2 accounted for above 50% of yield, which suggested M2 as the main fraction of F2 and thus was involved in anti-proliferating activity tests. Figure 3b shows the peak of purified M2 by preparative GPC. M2 is light yellow crystals with strong delicate flavor of *M. esculenta*, and is more water-soluble than M1.

### 2.4. Chemical Analysis

Chemical analysis of M2 hydrolysates indicated the monosaccharide components of a sample by GC compared with standard monosaccharides. Results showed M2 contains xylose (Xyl), glucose (Glc), mannose (Man), rhamnose (Rha) and galactose (Gal) in the ratio of 5.4:5.0:6.5:7.8:72.3 (Table 4), as well as 4.1% sulfate and 8.6% uronic acid. GPC spectrum suggested that M2 is homogeneous. Currently, the position of the sulfate group in M2 is unclear and needs further research.

It was provn that the presence of uronic acid, Gal and β-type glycosidic linkage contributed to antitumor activity [16]. Thus, we studied the anti-proliferating activity of M2 against tumor cells according to the chemical analysis.

### 2.5. FT-IR and NMR Spectra

**Figure 4:**
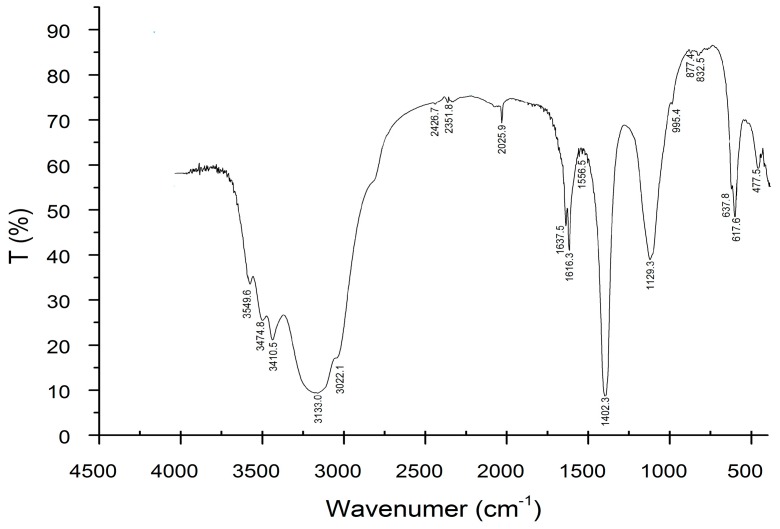
Fourier-transform infrared (FT-IR) spectrum of M2.

The signals of typical groups in M2 were shown in the FT-IR spectra (Figure 4). The absorptive peak at 3410.5 cm^−1^ indicated the presence of inter- and intramolecular hydrogen bonds, featuring a hydroxyl stretching vibration. The peak at 2351.8 cm^−1^ showed a C–H transiting angle. The broad high-intensity peak at 1402.3 cm^−1^ could be assigned to the deforming vibration of C–H in skeleton of galactans [17]. The strong peak at 1129.3 cm^−1^ indicated the existence of pyranose [18]. These results present that M2 possesses the typical absorption peaks of polysaccharides. The absorption peak at 877.4 cm^−1^ was assigned to the 4-sulfate and 6-sulfate of d-galactose units [19]. Meanwhile, α-d-Gal had absorptive peaks at 839–810 cm^−1^, or α-d-Man had the weak absorption at 832.5 cm^−1^. These details would be revealed by NMR analysis. The absorption within 500–650 cm^−1^ was assigned to the skeletal modes of pyranose rings [20]. The C=O asymmetric stretching vibration at 1616.3 cm^−1^ indicates the existence of uronic acid [21], which was consistent with the chemical analysis.

**Figure 5:**
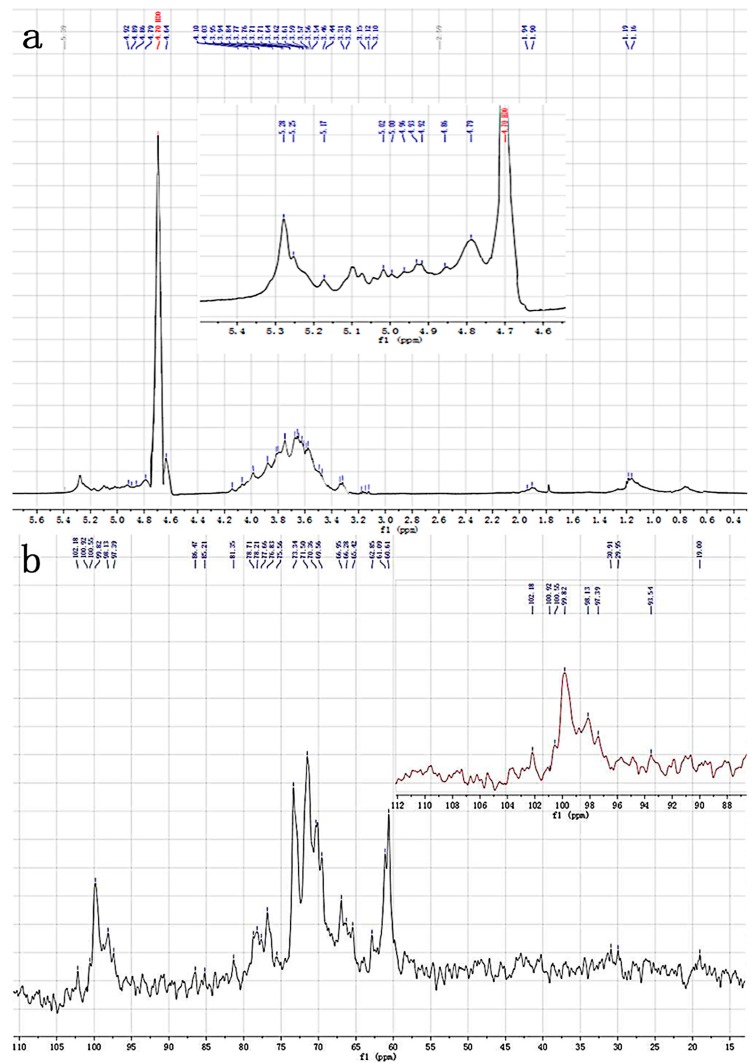
^1^H-nuclear magnetic resonance (NMR) (**a**) and ^13^C-nuclear magnetic resonance (NMR) (**b**) spectra of M2.

The ^1^H-nuclear magnetic resonance (NMR) and ^13^C-nuclear magnetic resonance (NMR) spectra of M2 are shown in Figure 5a,b, respectively. Most polysaccharides could be dissolved in deuterated water (D_2_O) and dimethyl sulfoxide (DMSO), which both were common solvents for polysaccharides in liquid-state NMR experiments. Additionally, NMR spectra were standardized with some internal standards (e.g., DMSO) and external standards (e.g., sodium 2,2-dimethy l-2-silapentane-5-sulphonate (DSS) and tetramethylsilane (TMS)). In practice, the response signals of internal standards were quite close to those of M2, so here we only adopted external TMS in the spectra.

The proton signals of M2 in the ^1^H-NMR spectrum were overlapped within δ 3.0–5.5 ppm, while the carbon signals in the ^13^C-NMR spectrum were overlapped within δ 60–110 ppm, so it was difficult to assign these signals. Therefore, we used the ^1^H- and ^13^C-NMR spectra to characterize the primary structures of typical polysaccharides in detail, and referenced some chemical shift patterns. As reported, Magdalena studied the NMR spectra of exo-polysaccharides and summarized the chemical shifts (in ppm relative to external DSS with a D_2_O signal at δ 4.40 ppm) of α-l-Rha*p*, α- and β-d-Glc*p*, and α-d-Gal*p* with different substituent positions [22]. In our study, the chemical shift of D_2_O was at δ 4.70 ppm (Figure 5a) considering only the influence of solvent D_2_O. Our ^1^H-NMR spectrum showed an increase of 0.3 ppm in the chemical shift.

The anomeric region of ^1^H between δ 4.93 and δ 5.28 ppm showed two characteristic signals of two α-type and one β-type glycosidic bonds [21]. The anomeric resonances at δ 5.10 and δ 5.28 ppm were due to (1→4)-linkedα-l-galactose-pyranose units [23]. This finding also indicated the anomeric carbon region on the ^13^C-NMR spectrum at δ 102.18, δ 100.92 and δ 100.55 ppm. Thus, together with the FT-IR spectrum, it was reasonable to conclude that the 1,3-link Glc residue was β-configuration, and the other Gal residue is α-configuration. It was speculated that β-1,3-Glc, α-l-Gal, and another α-type glycosidic linkage might exist in M2.

The downfield H-1 signals were typical of α-configurations in the pyranosyl series [24]. The resonances contained signals (C-1/H-1) at δ 100.55/5.28 and δ 98.82/5.25, corresponding to α-Manp. The other signals at δ 62.85, δ 61.09 and δ 60.61 corresponded to non-substituted *O*-6 of α-Manp units [25]. Meanwhile, the downfield C-1 resonances were typical of β-Xylp units. The signals (C-1/H-1) at δ 102.18/4.80 corresponded to β-Xylp units. Other resonances, such as those at δ 76.84, δ 71.5, δ 70.13, and δ 65.43 ppm, could be assigned to C-3, C-2, C-4 and C-5, respectively, of non-reducing end units of β-Xylp [26].

The signals of three glucan units (Table 5G–I) were assigned by comparing with the model-determined compounds (Table 5A–F) [28]. These model-determined compounds presented special carbon signals. Some glucans could be identified with the proton signals stated above. β-d-Glcp, α-l-Rhap and anhydro-α-l-Gal in Table 5G–I were determined from the model in Table 5A–F.

On the ^13^C-NMR spectrum (Figure 5b), the six carbon peaks between δ 60–100 ppm corresponded to β-d-glucan [29]; the peak at δ 103 ppm is assigned to the chemical shift of anomeric carbon in (1→3)-β-d-glucan [2]; and the peak around δ 100 ppm was attributed to the shift of C-1 in (1→3)-α-d-glucan. The β-glucan was conducive to antitumor activity [16].

The bioactivities of polysaccharides, especially antitumor activity, can be influenced by their monosaccharide composition, molecular mass [30] and chain conformation [31]. It is proven that the purified β-glucan-containing polysaccharides extracted from mushrooms can be applied for immunotherapy and cancer treatment in clinic [32].

### 2.6. Antitumor Activity in Vitro

**Figure 6:**
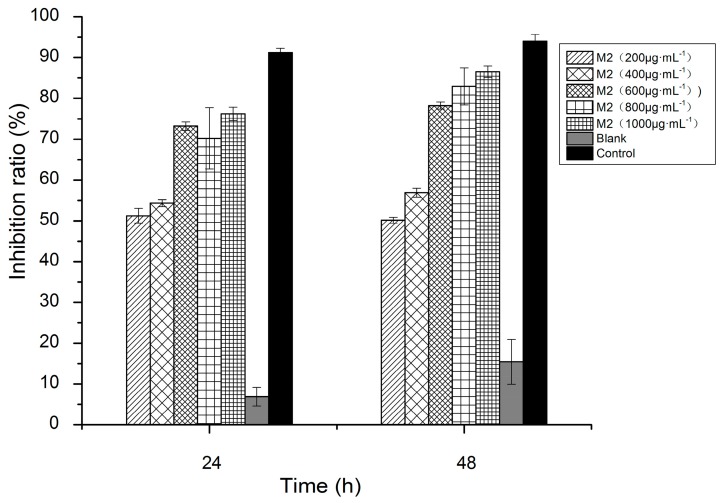
The antitumor activity of M2 on HT-29 cells after different incubation periods and at different concentrations in 3-(4,5-cimethylthiazol-2-yl)-2,5-diphenyl tetrazolium bromide (MTT) assay.

**Figure 7:**
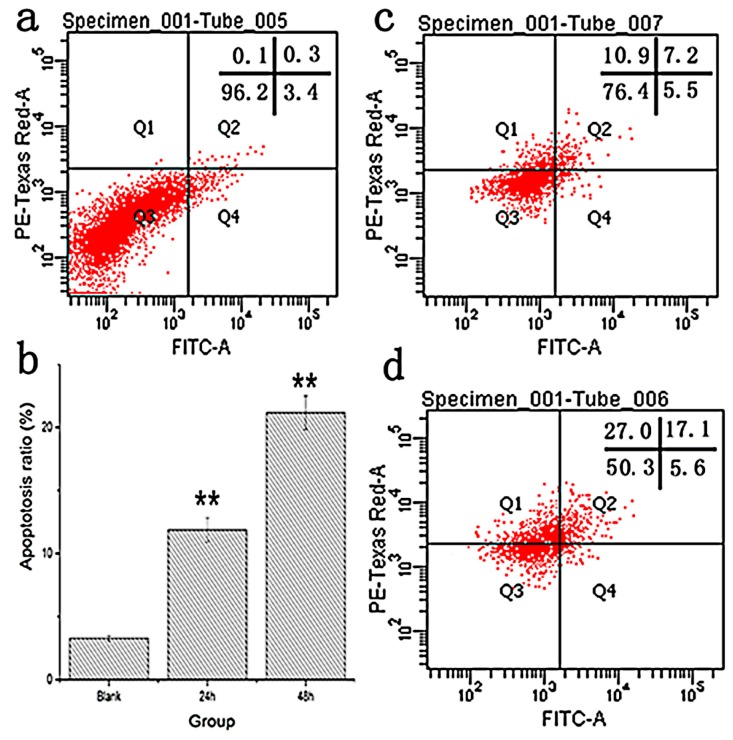
M2-induced apoptosis in HT-29 cells: (**a**) natural apoptosis of HT-29 cells without M2 (Blank) at 48 h on flow cytometry; (**c**,**d**) apoptosis of M2-treated cells at 24 and 48 h, respectively, on flow cytometry; and (**b**) bar graph summarizes the percentage of apoptosis. Data are expressed as mean ± SD of six replicate. **: *p* < 0.01.

M2 was selected for 3-(4,5-cimethylthiazol-2-yl)-2,5-diphenyl tetrazolium bromide (MTT) assay of its cytotoxicity against colon tumor cells (HT-29). In order to detect and distinguish the apoptotic and necrotic cells, we set a blank group that showed the natural mortality of HT-29 cells. Statistics analysis showed that the HT-29 cells in the blank group had a natural mortality of 6.09% after 24 h and of 19.05% after 48 h (Figure 6). The cytotoxic potential was tested over a wide concentration range from 200 to 1000 µg·mL^−1^ at two periods of 24 and 48 h. Even after a low-concentration (200 µg·mL^−1^) short-period (24 h) intervention, M2 still inhibited the growth of above 54.29% of HT-29 cells. However, the inhibitory activity of M2 on HT-29 cells was not significantly enhanced after 48-h when the M2 concentration exceeded 800 µg·mL^−1^ (*p* > 0.05) (Figure 7).

Tumor cells with high proliferative capacity can cause tumor deterioration. The signal pathways of apoptosis can profoundly affect cancer progression. Regarding the anti-proliferative effect of M2, we used Annexin V-FITC/PI staining to assay the cell apoptosis and cell cycle distribution. The apoptotic ratios were expressed as the proportion of apoptotic cells (Annexin V-positive). Compared to the blank group (Figure 7a), the percentages of apoptotic HT-29 cells treated with 800 µg·mL^−1^ M2 reached 12.9% after 24 h (Figure 7c) and 22.7% after 48 h (Figure 7d), which were associated with a concomitant increase of apoptotosis percentage. As shown in Figure 7b, the apoptotic ratio increased in a concentration-dependent manner after 48 h of M2 treatment, and it increased significantly from 12.88 ± 0.9657 to 22.64 ± 1.0326 as the intervention period from 24 to 48 h (*p* < 0.01). The results demonstrate that M2 could inhibit the growth of HT-29 cells via the induction of apoptosis. However, the toxicity of M2 to colon cancer cells is still unclear and needs to be studied in the future.

## 3. Discussion

Given its advantages over other conventional extraction methods (e.g., hot water extraction), PEF was selected to extract MEP. PEF treatment could improve the yield of MEP, which is exactly in accordance with some literature [10,11,14]. Besides, PEF extraction was operated without temperature rise [11]. Therefore, it caused less thermal degradation than heating extraction and decreased the change of *M*_W_s of polysaccharides. However, it can also induce degradation of polymer [12,33]. Since the efficiency and damage of PEF extraction on polysaccharide depend on the electric field intensity [34], medium intensity below 25 kV·cm^−1^ was chosen so as to decrease the degradation and increase the extraction ratio. It is really profound to choose probable parameters of PEF, which would be studied further.

Through separation and purification of polysaccharides, it is still hard to obtain purified fraction with less loss of target polysaccharide. We collected the M2 fraction from latter part of the peak in the preparative High-performance liquid chromatograph (HPLC) profile (Figure 3b), and abandoned the major forepart of M2. This selective collection ensures a higher purity of polysaccharide.

In early years, several ex-polysaccharides were isolated from *M. eculenta* [35]. Recently, endo-polysaccharides of *M. Esculenta* attracted attention from researchers for their biochemical activities [8,36]. One polysaccharide with the average *M*_W_ of 43,625 Da was extracted from *M. esculenta* by hot water (90 °C, 2 h), without detailed structural data from NMR or any activity, however [7]. The structure of anti-proliferative MEP in our study was elaborated by Fourier-transform infrared (FT-IR), ^1^H and ^13^C liquid-state nuclear magnetic resonance (NMR). Probably because of less-thermal extraction by PEF, the *M*_W_ of MEP was larger than theirs. The predominant polysaccharides (M2) in MEP separated into four fractions were cytotoxic to HT-29 cancer cells. This biochemical activity in accordance with glycosyl composition and linkages was mentioned before [37,38]. As reported, the ethanolic extract of cultured mycelium of *M. esculenta* was anti-tumoral against Dalton’s lymphoma ascite (DLA) and Ehrlich ascites carcinoma (ECA) cells [6]. Based on research on the antioxidative activity [39], the anti-colon-cancer activity of M2 on the proliferation of HT-29 cells was tested and found to be significantly time- and dose-dependent within 48 h. This finding suggests the potential therapeutic use of M2 in chemotherapy, and enriches the category of malignant cells. Our results only demonstrate the preliminary effect of M2 on HT-29 cells *in vitro*, but the preventive or inhibitory effect and suitable dose remain to be studied in tumor-bearing mice in the future. This study provides more information on chemical structural characterization of anti-proliferating and antitumor polysaccharides isolated from *M. esculenta.*

## 4. Materials and Methods

### 4.1. Materials and Instruments

*M. esculenta* was procured from MianYang Edible Fungi Center (Sichuan, China). A self-designed high-intensity PEF apparatus (2000–5000 Hz) was used.

### 4.2. Submerged Fermentation of M. esculenta

*M. esculenta* mycelia were cultured on a Potato Dextrose Agar medium (PDA) which contained (g·L^−1^): potato 100, sucrose 30, peptone 2, yeast extract 5, KH_2_PO_4_ 0.5 and MgSO_4_ 0.5 as well as 1000 mL distilled water. This medium was initially adjusted to pH 7.0. The *M. esculenta* mycelia were activated from a slant into a sterile Petri dish at 26 °C for 4 days on PDA, and then inoculated in 500-mL flasks at 26 °C for 5 days in a rotary shaker at 100 rpm. The as-obtained mycelia were washed with deionized water three times and lyophilized for 24 h.

### 4.3. M. esculenta Submerged Fermentation

The polysaccharides were extracted by PEF. Relevant indexes were calculated as follows:

(2) $$C=2\times \frac{\pi r^{2}l}{Q}\times f$$

(3) $$E=\frac{V_{pp}}{2l}$$

where *C*: pulse number; *Q*: flow velocity of sample (mL·min^−1^); *r*: radius of electrode (0.5 mm); *l*: length of electrode (1.5 mm); *f*: frequency (Hz); *E*: electric field intensity (kV·cm^−1^); and *V_pp_*: input voltage.

The PEF extraction conditions were optimized by the Box–Behnken design (BBD) of the respond surface methodology (RSM) with three variables each at three levels (Table 6). The experimental plan consisted of 17 trials and each dependent response was expressed as the mean of three replications. The second-order polynomial coefficients were calculated and analyzed on Design Expert 8.0.5 (Stat-Ease Inc., Minneapolis, MN, USA). The model was statistically tested via analysis of variance (ANOVA). Meanwhile, the effects of three key independent variables including electric field intensity (15, 20, 25 kV·cm^−1^), pulse number (4, 6, 8), and material-to-liquid ratio (1:20, 1:30, 1:40 g·mL^−1^) on the MEP yields were investigated separately. After PEF-based extraction, each sample was centrifuged at 4000 rpm for 20 min. Then after filtration, the filtrate was added with three volumes of ethanol and the resulting mixture was allowed to stand at 4 °C for 12 h. Finally, the precipitates were deproteinized with the sevage method for 24 h. The contents of polysaccharides were determined with the anthrone-sulfuric acid method [40] with glucose as the standard.

### 4.4. Purification of MEP Fractions

The crude polysaccharides at a concentration of 40 mg·L^−1^ were purified on a column chromatograph (GE Healthcare Bio-Sciences AB, Pittsburgh, PA, USA) with aSephadex G-100 column (2.0 × 60 cm). The column was eluted with deionized water at the flow rate of 12 mL·h^−1^.

### 4.5. Chemical Analysis

About 10 mg of MEP sample was hydrolyzed with 2 mol·L^−1^ trifluoroacetic acid (TFA) (100 °C, 8 h). In methylation analysis, the sample was acetylated and then analyzed on an HP5890 gas chromatograph (GC, HP Company, Ramsey, MN, USA) equipped with a capillary column (30 m × 0.25 mm). The GC conditions were: helium as the carrier gas; temperature program initially at 140 °C for 10 min, heated at 10 °C/min to 240 °C, then held there for 10 min; and split ratio = 20:1. The standard sugars (rhamnose (Rha), arabinose, xylose (Xyl), mannose (Man), fucose, galactose (Gal), and glucose (Glc)) were also converted to their acetylated aldononitrile derivatives and analyzed in the same way. The contents of monosaccharides were quantified from the peak areas using the response factors of standard monosaccharides. Total uronic acid content was analyzed by an m-phenylphenol method with glucuronic acid as the standard [41]. Sulfate content was measured according to the method by Dodgson [42].

### 4.6. Determination of Molecular Weight (M_W_)

Both homogeneity degrees and molecular weights of MEP fractions were determined by a gel permeation chromatograph (GPC, Shimadzu LC-10A, Tokyo, Japan) equipped with a TSK-GEL G 3000 PW_XL_ column (7.8 m × 300 mm). The conditions were: elution with ultrapure water at a flow rate of 0.5 mL·min^−1^, 45 °C, 2.0 MPa and injection volume 20 µL for each run. During the elution, the polysaccharides were monitored by a refractive index detector. The column was pre-calibrated with standard glucosans (Sigma, Round Rock, TX, USA; *M*_W_: 2500, 21,400, 41,100, 84,400, and 133,800 Da). Each fraction was produced by preparative chromatography with the TSK-GEL G 3000 PW_XL_ column and eluted with ultrapure water at a flow rate of 0.5 mL·min^−1^.

### 4.7. Spectroscopic Methods

The polysaccharides were characterized on an IRPrestige-21 Fourier-transform infrared (FT-IR) spectrometer (Shimadzu) in the range 4000–400 cm^−1^ via the KBr disk method [43]. Each time, 2 mg of polysaccharides was added into 200 mg of KBr.

### 4.8. Nuclear Magnetic Resonance (NMR) Spectroscopy

A lyophilized sample was dissolved in D_2_O, and then its ^1^H- and ^13^C-NMR spectra were recorded on a Bruker Ascend 500 NMR spectrometer (Bruker Daltonik GmbH, Bremen, Germany). The ^13^C-NMR chemical shifts are expressed in parts per million (ppm) relative to external tetramethylsilane (TMS).

### 4.9. Anti-Proliferating Activityinvitro

The human colon cancer cell line HT-29 was maintained in Dulbecco’s modified Eagle’s medium (DMEM; 4.5 g·L^−1^ glucose) at 37 °C and 5% CO_2_ in a humidified incubator. The DMEM was supplemented with 10% fetal calf serum, 100 U·mL^−1^ streptomycin and 100 U·mL^−1^ penicillin.

The tumor cell viability was quantified via a modified 3-(4,5-dimethylthiazol-2-yl)-2,5-diphenyltetrazolium bromide (MTT) system (Sigma), which is based on the conversion of the tetrazolium salt 3-(4,5-dimethylthiazol-2-yl)-5-(3-carboxymethoxyphenyl)-2-(4-sulfophenyl)-2-tetrazolium to formazan catalyzed by mitochondrial dehydrogenase [44]. The living cells turned to measurable bluish violet.

The HT-29 cells were inoculated for 24 h on a 96-well cultivation plate (100 µL·well^−1^) at a density of 1 × 10^6^ cells·mL^−1^ and at 37 °C with 5% CO_2_. After that, the cells were added with different concentrations of the target MEP fraction (200, 400, 600, 800, or 1000 µg·mL^−1^; this fraction was found to be M2) and cultivated for 24 or 48 h. MTT was added to the cultures at a final concentration of 0.5 mg·mL^−1^. The supernatant cells were removed by centrifugation, and then added with 200 µL of dimethyl sulfoxide (DMSO). The optical density (OD) at 570 nm [9] was measured on a micro-plate Reader (Bio-Rad Laboratories, Hercules, CA, USA). The ODs of tested cells were compared with the blank control group (HT-29 cells without addition of M2) and a positive control group (HT-29 cells treated with 10 µg·mL^−1^ 5-fluorouracil). The antitumor activity of a tested sample was expressed as the inhibition ratio (IR) as follows:

(4) $$\text{IR}\;\left(\%\right)=\left(1-\frac{A_{1}}{A_{0}}\right)\times 100\%$$

where A_1_: OD of an M2-treated sample; and A_0_: OD of an untreated sample.

### 4.10. Statistical Analysis

Data were expressed as means ± standard deviation (SD) for triple determinations. The differences among means were assessed via ANOVA and least significant difference (LSD) tests. The significance level was set at *p* < 0.05.

## 5. Conclusions

Polysaccharides consist of monosaccharide residues joined by glycosidic linkages. Compared with proteins and nuclear acids, polysaccharides offer the highest capacity for kinds of biological information because they have greatest variability of molecular structure [45]. Such variability gives great complexity and makes them difficult to extract without damaging the molecular structure. Compared with heat extraction, PEF degrades polysaccharides less.

Only the preliminary effect of M2 on proliferation of HT-29 cells *in vitro* was studied in this recent work, and the mechanism and suitable dosage remain to be studied in tumor-bearing mice in the future.

## Figures and Tables

**Table 1:** Experimental design of code levels and factors in respond surface methodology (RSM) by Box–Behnken design (BBD) matrix. MEP: *Morchella esculenta* polysaccharide.

Run Order	X_1_	X_2_	X_3_	MEP (µg·mL^−1^)
1	−1	1	0	50.21
2	0	1	1	38.15
3	0	0	0	55.62
4	1	1	0	39.15
5	0	−1	−1	39.27
6	−1	0	−1	50.07
7	1	−1	0	35.98
8	0	0	0	53.08
9	1	0	−1	37.23
10	0	1	−1	46.96
11	0	0	0	54.92
12	0	−1	1	35.51
13	−1	0	1	37.95
14	0	0	0	52.66
15	1	0	1	36.07
16	0	0	0	50.13
17	−1	−1	0	36.04

**Table 2:** Results of analysis of variance (ANOVA).

Source	Sum of Squares	df	Mean Square	*F* Value	*p*-Value Probability > *F*	Significance
Model	932.2642	9	103.5849	27.53076	0.0001	significant
X_1_	83.4632	1	83.4632	22.18282	0.0022	**
X_2_	95.70361	1	95.70361	25.43607	0.0015	**
X_3_	83.52781	1	83.52781	22.19999	0.0022	**
X_1_X_2_	30.25	1	30.25	8.039835	0.0252	*
X_1_X_3_	30.0304	1	30.0304	7.98147	0.0256	*
X_2_X_3_	6.375625	1	6.375625	1.694512	0.2342	
$X_{1}^{2}$	166.5724	1	166.5724	44.27157	0.0003	**
$X_{2}^{2}$	186.046	1	186.046	49.44725	0.0002	**
$X_{3}^{2}$	186.8866	1	186.8866	49.67067	0.0002	**
Residual	26.33761	7	3.762515			
Lack of Fit	7.825525	3	2.608508	0.563634	0.6673	not significant
Pure Error	18.51208	4	4.62802			
Cor Total	958.6018	16				

df: degree of freedom; * : *p* < 0.05; **: *p* < 0.01.

**Table 3:** Yields and relevant molecular parameters of four peaks of F2 in gel permeation chromatography (GPC) analysis.

Fractions	Parameters								
	Retention Time (min)	Yield (%)	*M*n (Da)	*M*_W_ (Da)	*M*z (Da)	*M*v (Da)	*M*_W_/*M*n	*M*v/*M*n	*M*z/*M*_W_
M1	13.054	25.575	198,950	222,344	256,908	222,344	1.11758	1.11758	1.15545
M2	14.205	52.119	74,633	81,835	89,127	81,835	1.09651	1.09651	1.08909
M3	20.308	13.929	413	428	443	428	1.03685	1.03685	1.03495
M4	21.286	8.377	183	192	201	192	1.05083	1.05083	1.04301

*M*n, *M*_W_, *M*z, and *M*v are present number, weight, z-average, and viscose molecular weight, respectively; *M*_W_/*M*n means poly dispersity ratio.

**Table 4:** Chemical analysis of M2 of MEP.

Fraction	Total Sugar (%, *w*/*w*) ^a^	Sulphate (%) ^a^	Uronic Acid (%) ^a.b^	Monosaccharides Composition (%)				
M2	77.8	4.1	8.6	Xyl	Glc	Man	Rha	Gal
				5.4	5.0	6.5	7.8	72.3

^a^ Percentage of the dry weight of F2(%, *w*/*w*); ^b^ the monosaccharide composition was detected by gas chromatography (GC) analysis (molar ratio).

**Table 5:** Chemical shifts (proton/carbon) of monosaccharide in M2 compared with model-determined compounds.

NO.	Sugar Residue	^13^C						Solvent	Ref.
		1	2	3	4	5	6		
A	→3)-β-d-Glc*p*-(1→	106.38	76.49	84.73	70.95	78.17	63.53	D_2_O(DSS)	[22]
B	→3)-α-l-Rha*p*-(1→	104.28	72.23	80.18	72.80	71.65	18.93	D_2_O(DSS)	
C	[α-Glc(1→3)]n	101.3	72.2	83.2	71.7	73.7	62.2	D_2_O	[27]
D	[β-Glc(1–2)-]n	102.7	83.1	77.0	69.3	76.1	61.4	D_2_O	
E	→3)-β-d-Gal-(1→	102.4	70.2	82.2	68.8	75.3	61.4	DMSO	[23]
F	→4)-3,6-anhydro-α-l-Gal-(1→	98.3	69.9	80.1	77.4	75.7	69.4	DMSO	
G	→3)-β-d-Glc*p*-(1→	102.18	76.83	81.35	70.36	78.21	62.85	D_2_O	M2 spectra in Figure 5b
H	→3)-α-l-Rha*p*-(1→	100.92	71.50	80.36	73.34	71.50	19.00	D_2_O	
I	→4)-3,6-anhydro-α-l-Gal-(1→	98.13	69.56	80.0	77.66	75.73	68.25	D_2_O	

**Table 6:** Coding table and selected values (obtained from previous studies) for experimental factors and levels in response surface method (RSM) for pulsed electric field (PEF) optimization.

Symbol	Code Levels	Factors		
		Electric Field Intensity (kV·cm^−1^)	Pulse Number	Material to Liquid Ratio (g·mL^−1^)
X1	−1	15	4	1:20
X2	0	20	6	1:30
X3	1	25	8	1:40

## Abbreviations

PEF	pulsed electric field
MEP	*Morchella esculenta* polysaccharide
PDA	Potato Dextrose Agar medium
BBD	Box–Behnken experimental design
RSM	respond surface methodology
GPC	gel permeation chromatograph
FT-IR	Fourier-transform infrared
NMR	Nuclear magnetic resonance
*M*w	molecular weight
ANOVA	analysis of variance
